# Questionnaire for Assessing Social Contacts of Nurses Who Worked with Coronavirus Patients during the First Wave of the COVID-19 Pandemic

**DOI:** 10.3390/healthcare9080930

**Published:** 2021-07-23

**Authors:** Matea Dolić, Vesna Antičević, Krešimir Dolić, Zenon Pogorelić

**Affiliations:** 1 Department of Health Studies, University of Split, 21000 Split, Croatia; vesna.anticevic@ozs.unist.hr; 2 Department of Diagnostic and Interventional Radiology, University Hospital of Split, 21000 Split, Croatia; kdolic79@gmail.com; 3 School of Medicine, University of Split, 21000 Split, Croatia; zpogorelic@gmail.com; 4 Department of Pediatric Surgery, University Hospital of Split, 21000 Split, Croatia

**Keywords:** COVID-19, nurses experience, social distancing

## Abstract

(1) Background: The aim of the present study was to develop and validate the psychometric characteristics of a scale measuring nurses’ experiences working with COVID-19 patients. (2) Methods: The participants were 180 Croatian nurses who worked in departments with COVID-19 patients, with a mean age of 36.8 years (ranging from 20 to 48). Research was conducted from March to June 2020. For the purpose of constructing the scale, 10 statements were developed. Factor analysis was used to determine the factor structure and construct validity of the scale. (3) Results: The scale consisted of nine statements divided into a three-factor structure: factor I—stigmatization and mistrusting (four items), factor II—social distancing (four items), and factor III—fear of infection (two items). Cronbach α was calculated to confirm the reliability of the scale: factor I—α = 0.80, factor II—α = 0.76, and factor III—α = 0.70. (4) Conclusion: The nurses’ pandemic-related experiences scale showed good psychometric properties and can be applied in future research as a standardized tool for measuring health care workers’ experience during COVID-19 or other infectious crises.

## 1. Introduction

The COVID-19 pandemic caused by new coronaviruses represents a major global public health care problem that is causing changes in the current way of life and work in all segments of society. The Covid-19 pandemic is progressively being regarded as a social problem rather than just an infectious disease. There are diseases that not only burden the medical system but also instill increased tension in each individual by provoking social stigma [1]. Health care professionals, including nurses around the world, have shown exceptional courage and professional morality in responding to the challenge of the pandemic in the International Year of Nursing and Midwifery [2,3]. The entire public, including health care professionals, are confronted daily with images and reports of a health care system collapse due to the COVID-19 virus pandemic, especially in Italy and Spain during the first wave of pandemic, but also around the world [4]. There was fear of infection and the possibility of transmission from family members and friends, but also fear of a lack of COVID-19 protective clothing for the health workforce and equipment to treat patients [5]. Staff may be concerned about their own risks from exposure to a new pathogen, or the risk that they might infect family or friends. These concerns can be particularly acute when the etiology and outcomes from a new virus are not well understood [6]. Data on the large number of sick health care workers in Italy and Spain—especially doctors and nurses, with over 100 deaths—further added to the unrest and concern among health care staff. Furthermore, the results of research conducted on health care workers who worked with COVID-19 patients in Wuhan, China, showed that health care workers reported symptoms of anxiety, depression, and insomnia. Also, in comparison with health care workers who worked with non-COVID-19 patients, nurses that worked “on the front lines” with COVID-19 patients showed negative outcomes of mental health [7]. Another piece of research on the psychological and mental impact of COVID-19 disease on medical staff and the general population has identified risk factors for anxiety and depression, including female gender, nurse occupation, lower socioeconomic status, high risk of COVID-19 virus infection, and social isolation [8].

A review of the literature did not find research using a standardized questionnaire to assess the socializing (at work and out of work) of nurses who worked with coronavirus patients during the first wave of the pandemic. The aim of this study was to develop and validate psychometric characteristics of a questionnaire designed to measure nurses’ experiences working with COVID-19 patients.

## 2. Materials and Methods

A correlation cross-sectional design was used in this study. The research was conducted online on a suitable sample of 180 nurses working in the health care system of the Republic of Croatia with regards to the COVID-19 virus pandemic. The survey was conducted via the Google docs platform, and was available to all nurses who used the Facebook group of nurses, and they responded to the questionnaire by clicking on the appropriate link. The inclusion criteria were the employment of a nurse in the Croatian health care system and providing health care services to the COVID-19 positive patients from March to June 2020. The exclusion criteria were the employment of a nurse outside the Croatian health care system, providing health care services to the COVID-19 negative patients, and a positive history of previous psychological problems. Subjects were of both sexes (female = 167, male = 13) and aged 20–48 years, with a mean age of 36.8 ± 15.5 years. The results analyzed in this study are the part of a broader study of the effect of ward work with patients with COVID-19 on a nurse’s mental health, in which a series of standardized mental health assessment questionnaires were used.

All participants gave their informed consent to present the data in the submitted manuscript by accepting the click of a button before taking the online tests. Participants completed the questionnaire on their own, which lasted approximately 5 min. Only data obtained based on responses to the scale of experiences associated with the pandemic nurses were used in this study. Participation was voluntary and completely anonymous, and the completion rate was 100%.

### 2.1. Instruments

A scale named “Nurses’ pandemic-related experiences questionnaire” was constructed for the purposes of this research. The scale consists of 10 statements examining the different experiences of nurses working in COVID-19 departments. Participants were asked to respond on a scale from 1 (does not apply to me at all) to 5 (fully applies to me). The total score of each participant is expressed as the final sum of responses to each statement [9].

Taking into account the specificity of pandemic-related professional experiences in comparison with non-pandemic working circumstances, the statements were compiled based on a study of literature sources on the most frequent stressors of health care providers during pandemics [10,11,12,13,14,15]. The questionnaire was also accompanied by items compiled based on an interview with three experienced nurses who were working in a COVID-19 department during the “first wave” of the pandemic about what was most stressful to them. Nurses expressed their perceptions of feelings, behaviors, and socializing during the first wave pandemic “lock-down”. In this way, an initial list of 10 stressors was obtained, the frequencies of which were estimated by 180 nurses who participated in this study. In order to examine the psychometric properties of the questionnaire, we performed a factor analysis, where one item had a saturation less than <0.5 and was thus excluded from further analysis. Scree plots were examined and enabled a three-factor model solution. The first factor (Stigmatization and misunderstanding) explained 45.70% of the variance, the second factor (Social distancing) explained 15.56% of the variance, and the third factor (Fear of infection) explained 10.71% of the variance. The final version of the questionnaire consists of 9 items. The results of factor analysis and reliability measures are explained in detail in the Results section.

The data collected from the questionnaire were entered into Microsoft Excel spreadsheets according to a previously prepared code plan.

### 2.2. Statistical Analysis

Data were recoded, sorted, and prepared for analysis using the SPSS version 26.0 software package (IBM Corp, Armonk, NY, USA). There was no missing data in the dataset. For the purpose of data processing, descriptive statistics were used to calculate means and standard deviations. To identify psychometric properties of the scale, the Principal Axis Factoring with Promax rotation method was used, including scree plots. The internal consistency and reliability were measured by McDonald’s ω and Cronbach’s α. The suitability of data for structure detection was verified using Kaiser–Meyer–Olkin (KMO) and Bartlett’s tests. The Kolmogorov–Smirnov (KS) test was used to examine the normality of distribution. As a part of statistical analysis, we also checked skewness and kurtosis to determine whether the data were heavy-tailed or light-tailed relative to a normal distribution. The results are presented in tables in the Results section. The α-error level was set to 0.05.

## 3. Results

### 3.1. Factor Structure

The Kaiser–Meyer–Olkin statistic proved the satisfying sampling adequacy of the data (KMO = 0.80), enabling the factor analysis. The Bartlett’s test of sphericity was significant (x2(21) = 676.77, *p* < 0.001).

An analysis of the main components with the Promax rotation method was performed and a three-factor structure was disclosed (Table 1). The Principal Axis Factoring extraction method was used to determine item saturations by each factor. Only one item (“When we were on shift, we had problems with food delivery because the restaurant staff did not want to deliver food to a department with infected persons”) had saturations <0.50 and was thus excluded from further analysis. The total score was recalculated with the remaining 9 items.

The first factor reflects feelings of stigma and misunderstanding that nurses had while working in a department with COVID-19 patients; this factor was named “Stigmatization and misunderstanding”. The second factor describes actual or planned distancing/avoidant behaviors of nurses in order to protect significant others; this factor was named “Social distancing”. The third factor describes nurses’ fears of infecting oneself or loved ones; this factor was named “Fear of infection”.

Descriptive indicators (means and standard deviations) for each statement are shown in Table 2.

### 3.2. Internal Consistency

The values of the internal reliability of both the items and the factors are shown in Table 3. All coefficients range from 0.81 to 0.88 for both measures, indicating satisfactory internal consistency of the items and extracted factors.

### 3.3. Testing for Normality

The Kolmogorov–Smirnov test indicated that the distributions within all three factors were not normally distributed (*p* < 0.001) (Table 4).

Additionally, the distributions of the three factors were examined for skewness and kurtosis (Table 5). The skewness of distributions indicate a moderate shift to the left. Furthermore, most kurtosis values are less than zero, showing platykurtic distribution with the central peak being lower and broader, as well as fewer values close to the mean.

Since most of the values for asymmetry and kurtosis ranged between −2 and +2, it can be considered acceptable in order to prove normal univariate distribution [9].

## 4. Discussion

Numerous studies have been conducted to investigate public perceptions of health care workers during the COVID-19 virus pandemic [1,3,5,7,8]. However, insufficient research has been conducted on health care professionals on stigmatization and misunderstanding, social distancing, and fear regarding the pandemic and their exposure to it.

The most important result of this study is that the scale of pandemic-related experiences of our nurses showed good psychometric properties. The validation results of the 10-particle scale in this paper point to a three-membered structure: Stigmatization and misunderstanding (four items), Social distancing (four items), Fear of infection (two items) Each of these three factors poses a risk of developing psychological and physical consequences from performing work and providing health care to patients with COVID-19.

### 4.1. Stigmatization and Misunderstanding

Being the target of stigmatization places individuals under great pressure. Stigma is a common phenomenon in the prevalence and spread of infectious diseases. It leads to negative emotions among the stigmatized, including stress, anxiety, sadness, and even some physical reactions [14,15,16,17,18].

Stigma leads to a social misunderstanding of risk and extreme fear amongst members of society, which is accompanied by a disproportionate allocation of health care resources by politicians and health care professionals [16,17].

Public exposure to dramatic images of deaths caused by the COVID-19 pandemic from Italy and other countries and dramatic news reporting the number of infected and deceased doctors and nurses has led the public to assess personal risk of infection through contact with health care professionals [4,14]. In a survey conducted in 2020, more than a third of respondents thought that health care workers were COVID-19 positive, almost half of respondents (47%) said they did not want to be near health care workers caring for COVID-19-positive patients, they had unrealistic attitudes about the danger of contact with health care workers and felt that they should even be banned or prevented from contacting their family members (31%) in order to prevent the possibility of spreading the infection [8]. Stigmatization creates an unnecessary burden on the lives of health care professionals and can contribute to the development of mental problems [9]. Confusion, misunderstandings, and the presentation of “false science” by sources deemed to be trustworthy are breeding grounds of stigma, as they evoke stereotypes, discriminatory behaviors, and prejudice [11,12,13]. Our results are in the line with reports from around the world that doctors and other health care providers have been isolated from loved ones because of anticipated risk of contamination, and have also been assaulted physically or emotionally due to fear and stigmatization [14]. This makes this already tough situation even more challenging, as the increased burden on medical staff’s mental health may negatively affect their functioning and resilience [15,16,17].

Further, stigmatization will arouse emotions and trigger the stress response or reaction mechanism. Due to the global nature of the COVID-19 pandemic, stigmatization has become a psychosocial phenomenon with a larger scope and more influence [18]. At present, while worldwide public health is facing difficulties, studies on the social-emotional burdens caused by stigmatization have real-life significance; thus, it is important to test the existing theories against the background of this global public health and security crisis [19,20].

The stigma needs to be addressed rigorously by professionals and health care providers as well as authorities [1,20].

### 4.2. Social Distancing

Avoiding socializing, or physical distancing, is considered an important measure to combat infection. Health care workers are also obliged to physically distance themselves from their colleagues in order to protect each other, causing them to go without the necessary social support, especially in these challenging times. The results of numerous studies during the COVID-19 pandemic have shown that health care workers’ relationships with family members and friends have changed. The measures of physical distancing and “lock down” have led to changes in social functioning, turning people towards their immediate family. The data obtained support the positive impact of the pandemic on the relations between close family members, especially parents and children [18,19,20,21]. In 2020, a survey of more than 4000 participants was conducted in Jordan, and the results suggested that the COVID-19 pandemic negatively affects the mental health of the Jordanian population, causing anxiety and depression in a significant portion of the population [22]. Social relationships and connections allow individuals to regulate their feelings, cope with stress, and remain resilient during stressful situations. In contrast, loneliness and social isolation exacerbate stress and often result in negative effects on mental, cardiovascular, and immune health [23].

### 4.3. Fear of Infection

Health care professionals working in a high-risk area (triage wards, inpatients, and intensive care units with COVID-19-positive patients) have a higher risk of exposure to infection. The outbreak of the pandemic changed the work scenarios of health care workers; they are directly responsible for the process of caring for patients both with and without COVID-19, must constantly wear personal protective equipment (which further complicates the implementation of medical procedures) [10,21], and lack specific treatment guidelines [24]. Wuhan showed that 88% of health care workers were exposed to COVID-19 infection [25]. Such a high risk of exposure to infection, care for patients, and fear of exposing their family members and loved ones to infection leads to fear, anxiety, and stress among health care workers, which can result in mental strain and the development of significant psychological problems [26,27,28]. A multicenter cross-sectional study of more than 1,000 Chinese health care workers recorded an exceptionally high proportion of depression (50%), anxiety (45%), and insomnia (34%) [27]. In a another meta-analysis of 13 studies, a total of 33,062 respondents confirmed that a large number of health care workers had significant levels of anxiety, depression, and insomnia during the outbreak of the COVID-19 pandemic [28,29]. The prevalence rates of anxiety and depression were about 23%. A high proportion of health care workers reported mild symptoms of both depression and anxiety, while moderate and severe symptoms were less common [28,29,30,31]. Fear and anxiety appeared and decreased in the early stages of the outbreak, and depression, psychophysiological symptoms, and symptoms of post-traumatic stress appeared in the second stage and lasted for a long time, leading to a more severe picture of the situation [29,30]. Nurses in hospitals have shown higher levels of stress than other health care professionals because they are in direct and intensive contact with patients [31].

Kang et al. estimated the impact of the COVID-19 pandemic on the mental health of physicians and nurses in Wuhan, soon after the onset of the pandemic [32]. Interestingly, half of the health care population had received psychological support through materials available online or provided by media, one out of three had obtained paper-based psychological counselling (brochures, leaflets, or books), and approximately one out of five had received individual or group psychotherapy [32,33].

Those who had been placed in quarantine, worked in high-risk facilities, or had close contacts (friends or family members) affected by SARS-CoV-2 were at up to a three-fold higher risk of having severe post-traumatic stress symptoms [10].

### 4.4. Limitations

This study has a several limitations. The first limitation of this study is related to the relatively small sample size and the fact that it was conducted only in Croatia at the end of the first wave of the pandemic, when participants were already sensitized and had more information about the pandemic to protect against the spread of infection. Also, it was used only among nurses and no other health care workers.

The second limitation is related to the online convenient sampling method that was used in this study, which could result in a distribution asymmetry. We are aware of the limitations of this type of sampling, but we would like to emphasize that, at the time when the research was conducted, strict epidemiological measures of social distancing were present, and that was the reason why all research, including this one, was conducted online, on convenience samples.

The next source of bias is related to the applied methodology that disables determination of the constructive and predictive validity of the questionnaire. Our primary goal was to verify the applicability of the scale by testing specific stressors in nurses and to identify the factors underlying nurses’ responses to the questions. Furthermore, as it was noted in the Methods section, this questionnaire is part of a broader study examining emotional responses of health care professionals working with infected patients during a pandemic. In future research, in addition to this questionnaire, we will also use other standardized scales, where the correlation between this scale and similar measures will be examined. Therefore, this research serves as a pilot study aimed to determine the justification for further use of this questionnaire for Croatian nurses working with infected patients.

Future research should include all categories of health care workers, verify the influence of public stigma on other groups in social public crisis events, and deeply explore different types of emotional arousal mechanisms for different groups.

## 5. Conclusions

This research conducted on nurses has proven that nurses as a profession are extremely prone to the development of burnout syndrome and various behavioral disorders and diseases. The COVID-19 pandemic further burdened nurses and all health care workers. Fear and avoidance of health care workers during the COVID-19 virus pandemic is a widespread problem throughout the world, but it is still not sufficiently recognized and can therefore have long-term consequences for the health of nurses and the health of other health care professionals’ families. One of the possible reasons for interventions to identify and prevent these problems is the lack of structured scales to measure it. The pandemic-related experiences scale of our nurses has shown good psychometric properties and can be applied in future research as a standardized measurement tool not only for nurses’ but also other health care workers’ experiences during the COVID-19 crisis or while working with other infectious patients.

## Figures and Tables

**Table 1:** Factor structure of the nurses’ pandemic-related experiences questionnaire.

Items	Factor I Stigmatization and Misunderstanding	Factor II Social Distancing	Factor III Fear of Infection
% of Variance	45.70	15.56	10.71
Item 1	0.917	0.383	0.246
Item 2	0.785	0.407	0.347
Item 3	0.580	0.570	0.373
Item 4	0.539	0.486	0.312
Item 5	0.398	0.852	0.553
Item 6	0.419	0.816	0.395
Item 7	0.312	0.661	0.639
Item 8	0.253	0.483	0.842
Item 9	0.311	0.389	0.689

**Table 2:** Descriptive properties of items (n = 180).

Item	Mean	Standard Deviation	Number of Items
I felt that my neighbors avoided me when we met in a building, on the street, or in a store because of my work in the hospital.	3.28	1.35	
People close to me avoided me because of fear of exposing them to a possible infection.	3.23	1.43	
I felt that I could not talk to close people about my work because they would not understand me.	3.24	1.55	
I preferred spending free time with my colleagues because we were at the same risk of infection, so I didn’t feel afraid that I would infect them.	3.44	1.45	
*Factor I: Stigmatization and misunderstanding*	**3.30**	**1.14**	**4**
I avoided intimacy with my partner because of the possibility to exposing him/her to a possible infection.	3.98	1.27	
I spent less time with my family because of the possibility to exposing them to a possible infection.	3.58	1.46	
I considered having physical separation from my family while working in a department with infected patients.	2.89	1.58	
When we were on shift, we had problems with food delivery because the restaurant staff didn’t want to deliver food to a department with infected persons.	2.54	1.59	
*Factor II: Social distancing*	**3.49**	**1.23**	**4**
I was afraid I would get a COVID-19 infection.	3.39	1.28	
I was afraid I would pass the infection on to my family.	4.47	0.91	
*Factor III: Fear of infection*	**3.93**	**0.98**	**2**

**Table 3:** Internal consistency of the nurses’ pandemic-related experiences questionnaire.

If Item Excluded		
Item	McDonald’s ω	Cronbach’s α
**1.**	0.83	0.83
**2.**	0.84	0.83
**3.**	0.84	0.84
**4.**	0.83	0.83
**5.**	0.84	0.83
**6.**	0.83	0.82
**7.**	0.83	0.83
**8.**	0.85	0.84
**9.**	0.84	0.84
**Total score for Factor I**	0.85	0.85
**Total score for Factor II**	0.82	0.81
**Total score for Factor III**	0.88	0.88

**Table 4:** Kolmogorov–Smirnov test of normality.

	Statistic	df	*p*
Factor I Stigmatization and Misunderstanding	0.12	180	<0.001
Factor II Social Distancing	0.13	180	<0.001
Factor III Fear of infection	0.17	180	<0.001

**Table 5:** Skewness and kurtosis of the nurses’ pandemic-related experiences questionnaire.

Factor	Kurtosis	Skewness
Factor 1	−0.74	−0.35
Factor 2	−0.95	−0.29
Factor 3	0.65	−0.97

## Data Availability

The data presented in this study are available upon request of the respective author. Due to the protection of personal data, the data are not publicly available.
